# Transcriptomic Analysis in Alzheimer’s Disease Brain During Systemic Infection

**DOI:** 10.1002/alz.090793

**Published:** 2025-01-03

**Authors:** Giulia Pegoraro, Rebecca G. Smith, Lachlan Ford MacBean, Adam Smith, Delphine Boche, Ehsan Pishva, Katie Lunnon

**Affiliations:** ^1^ University of Exeter, Exeter United Kingdom; ^2^ University of Exeter, Exeter, Devon United Kingdom; ^3^ University of Southampton, Southampton United Kingdom; ^4^ Maastricht University, Maastricht Netherlands

## Abstract

**Background:**

The onset and progression of Alzheimer’s disease (AD) has been widely linked with inflammation both in the periphery and within the brain. Indeed, infections during life can increase the risk of developing dementia and the rate of cognitive decline in AD patients, with many AD sufferers ultimately dying with a systemic infection. One of the aims of this study is to investigate the molecular mechanisms involved in the brain’s response to systemic infections in AD through the analysis of gene expression changes.

**Method:**

The cohort of patients selected for the study is composed of 204 post‐mortem prefrontal cortex brain samples. The cohort is divided as follows: 58 samples affected by AD and 33 controls, all of whom died with a respiratory infection, and 60 AD patients and 53 controls who did not have an infection at the time of the death. Following RNA sequencing; differential expression analysis, weighted gene correlation network analysis (WGCNA) as well as cell‐type and pathway enrichment analysis were performed.

**Result:**

The results of the differential expression analysis indicate that 1185 genes were differentially expressed. The cell‐type enrichment analysis revealed a significant enrichment in microglia, fundamental cells in brain immune response. Pathway enrichment analysis on the differentially expressed genes reported that the top ten enriched pathways were all associated with immune response; examples include “regulation of myeloid leukocytes mediated immunity.” A module from WGCNA also exhibited a correlation with both AD and infection. The cell‐enrichment analysis on the genes in this module demonstrated enrichment in oligodendrocytes and astrocytes, which are reported to be involved in immune response.

**Conclusion:**

This analysis laid the foundations for further investigations into gene expression changes associated with AD and infection. Future work will include combining this data with miRNA sequencing, DNA methylation and genotyping data from the same individuals to gain a comprehensive overview of the underlying biological mechanisms.

